# Supplementary figures and images for: A neural circuit framework for economic choice: From building blocks of valuation to compositionality in multitasking
Source: Neuron. Author manuscript; Available in PMC 2026 May 10. (PMC13157655; doi:10.1016/j.neuron.2025.12.010)

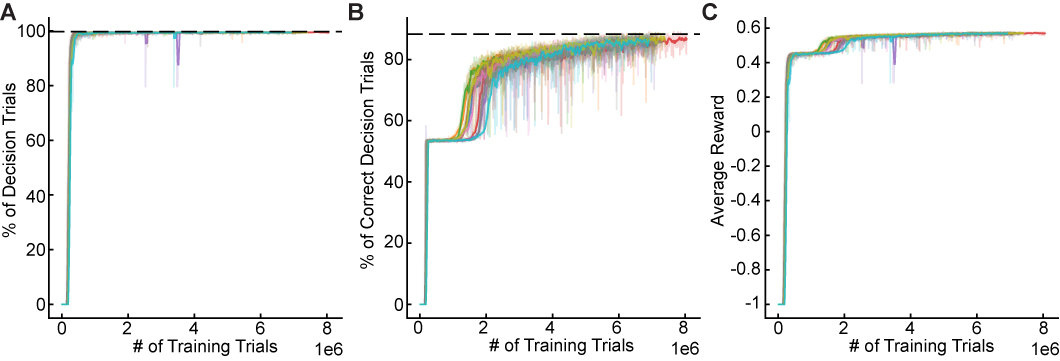

Supplement: Fig S1 [file NIHMS2167012-supplement-Fig_S1.jpg]

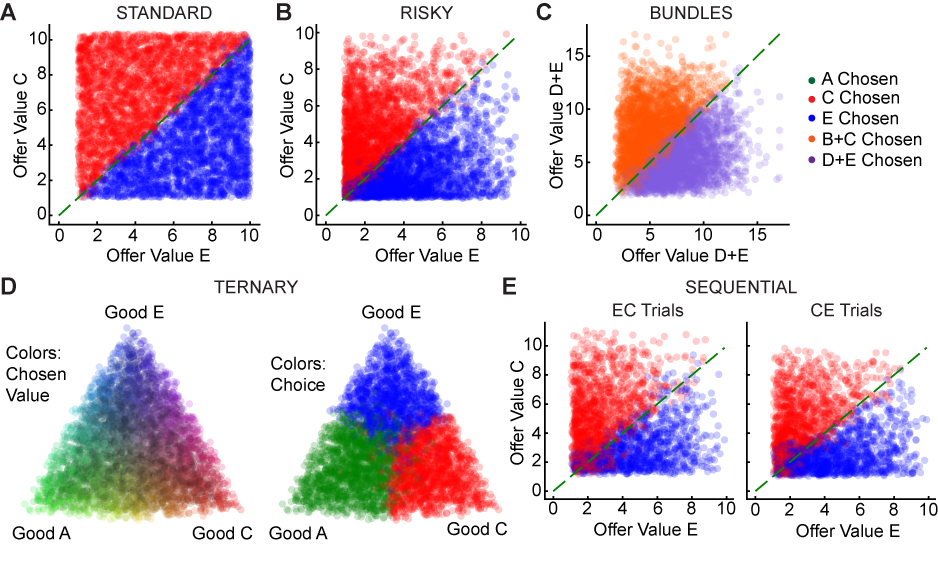

Supplement: Fig S3 [file NIHMS2167012-supplement-Fig_S3.jpg]

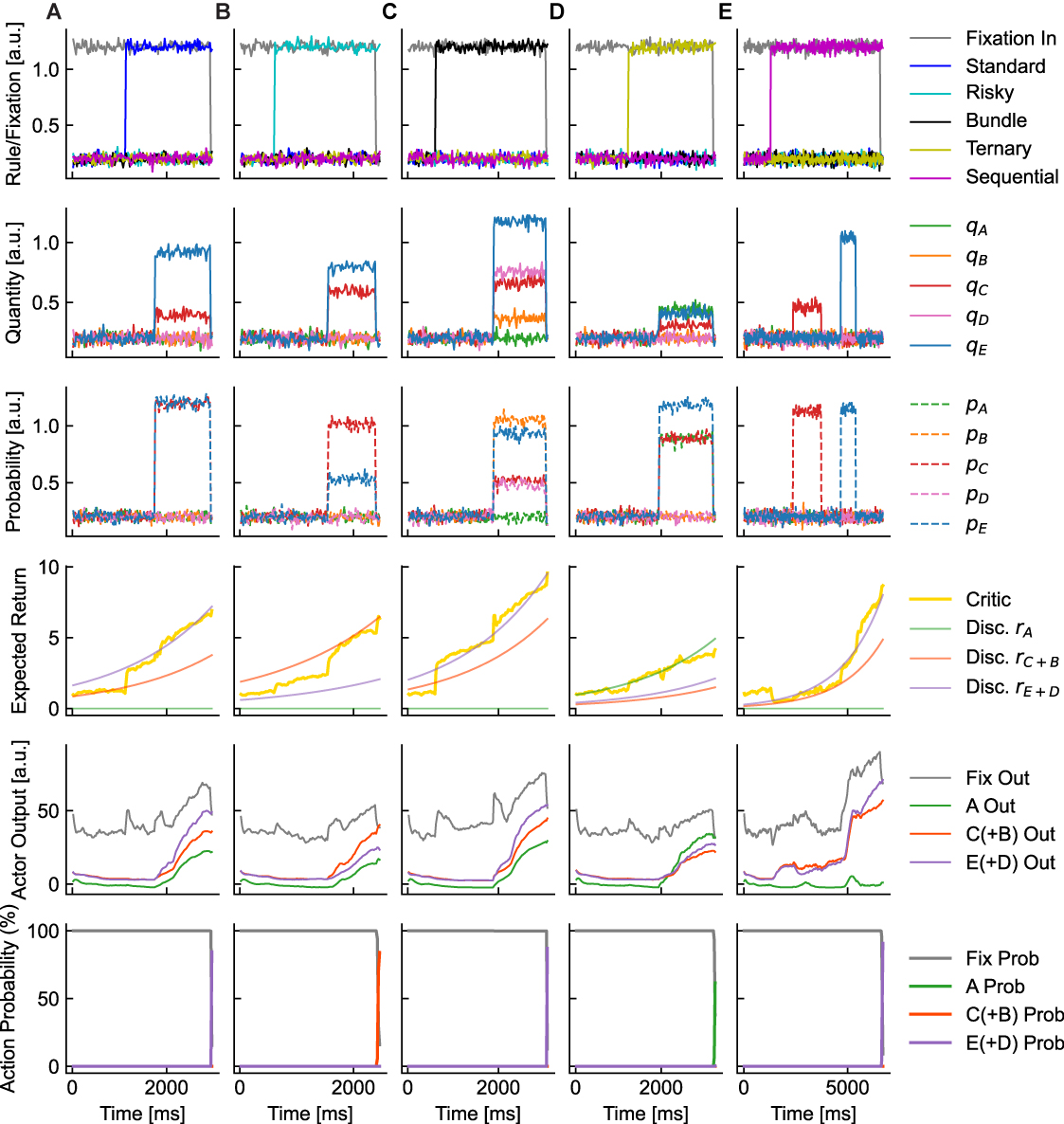

Supplement: Fig S2 [file NIHMS2167012-supplement-Fig_S2.jpg]

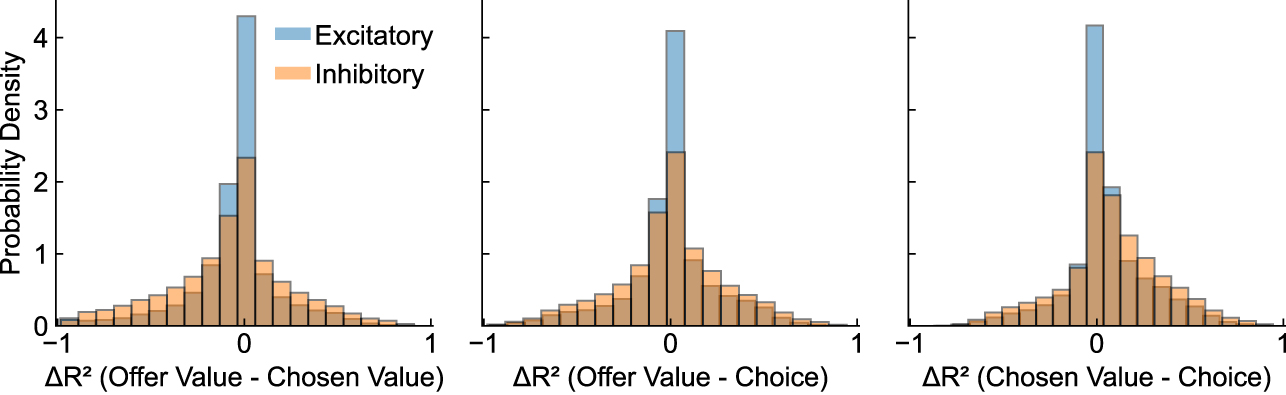

Supplement: Fig S4 [file NIHMS2167012-supplement-Fig_S4.jpg]

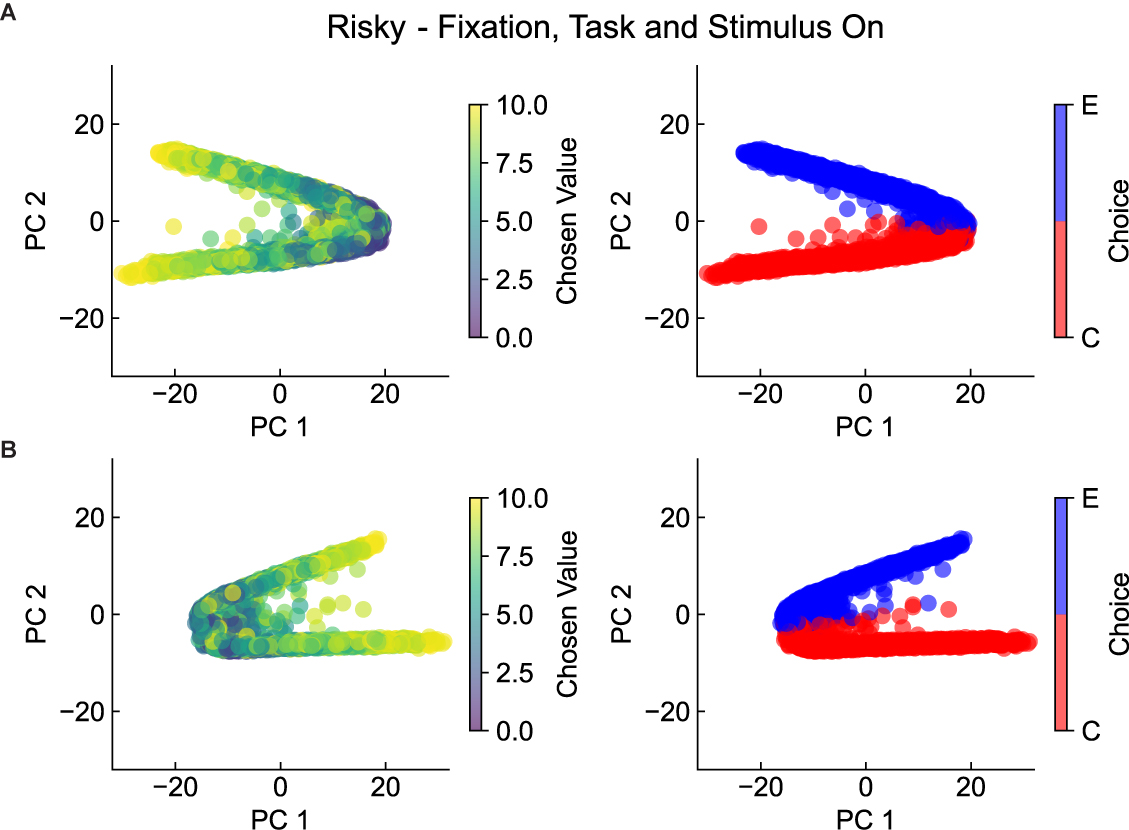

Supplement: Fig S5 [file NIHMS2167012-supplement-Fig_S5.jpg]

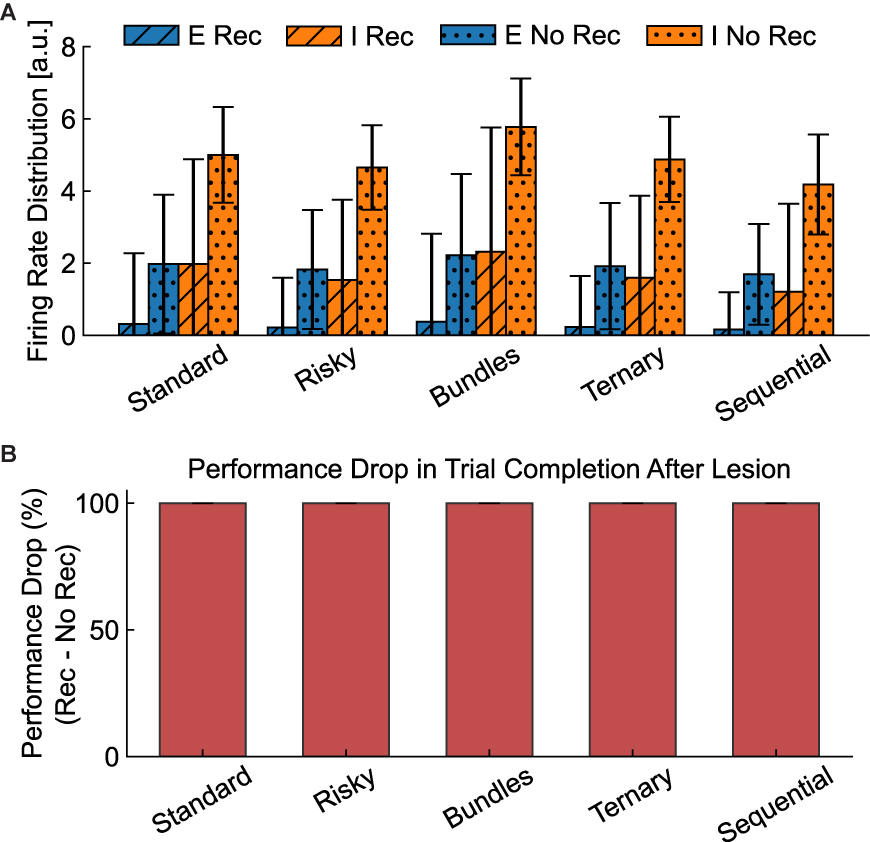

Supplement: Fig S6 [file NIHMS2167012-supplement-Fig_S6.jpg]

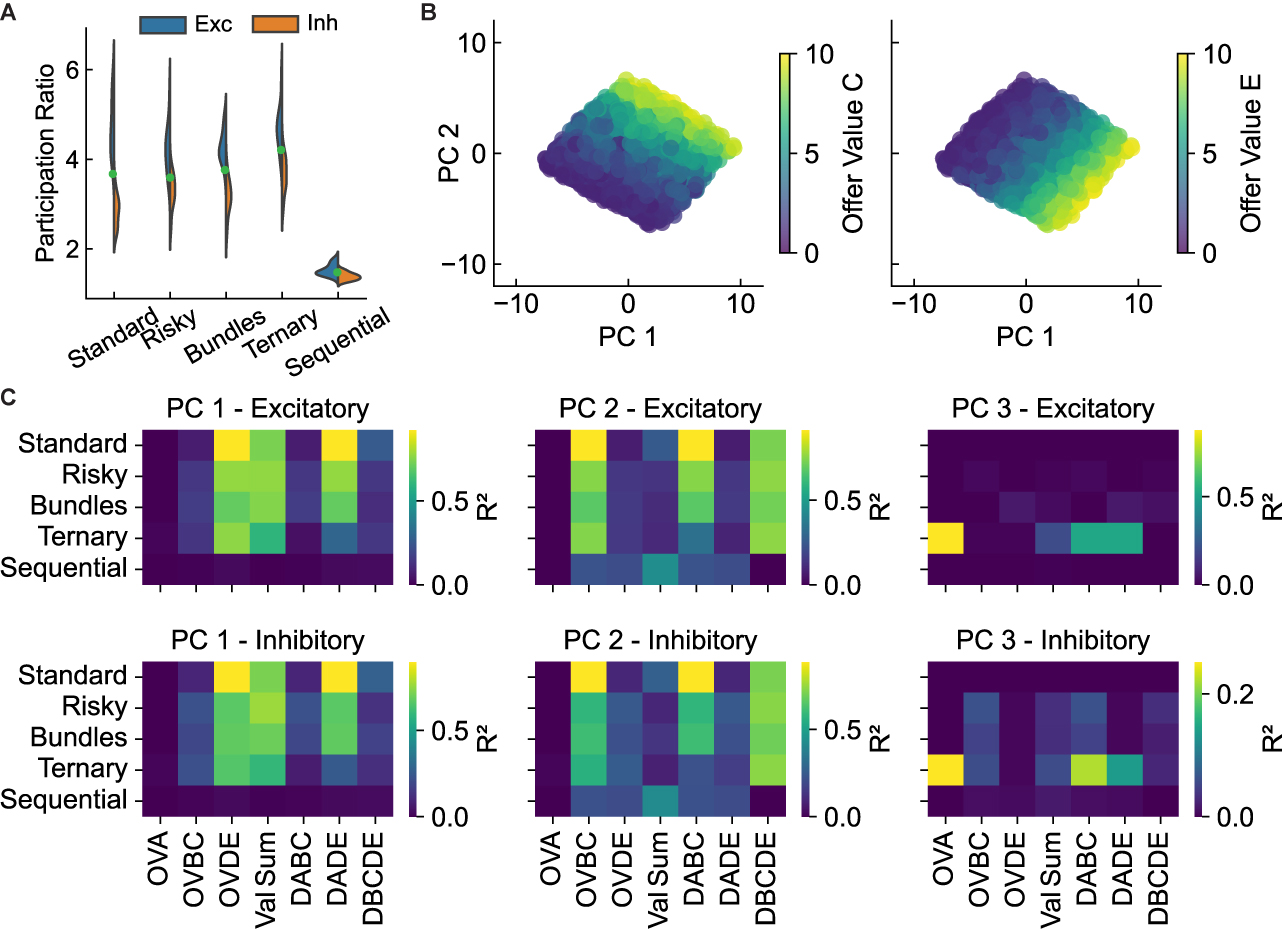

Supplement: Fig S7 [file NIHMS2167012-supplement-Fig_S7.jpg]

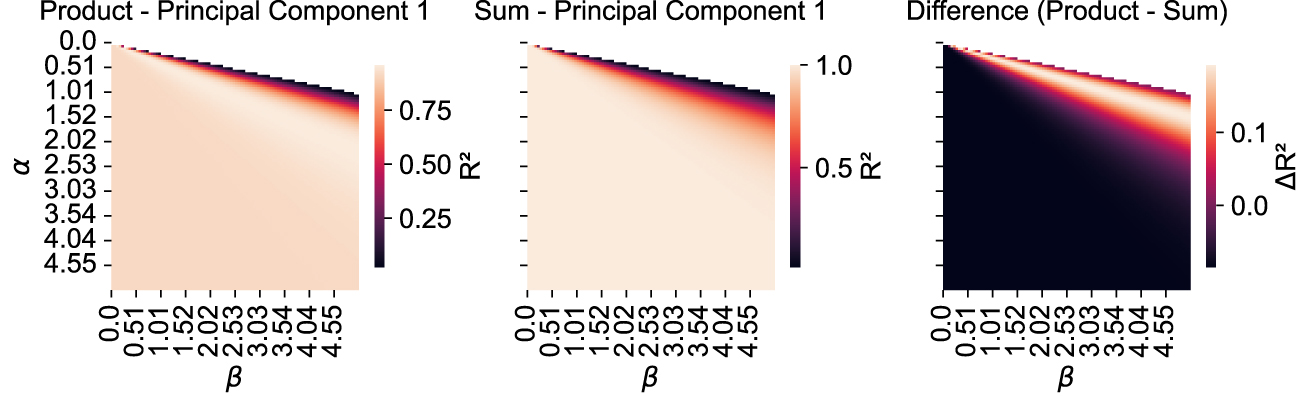

Supplement: Fig S8 [file NIHMS2167012-supplement-Fig_S8.jpg]

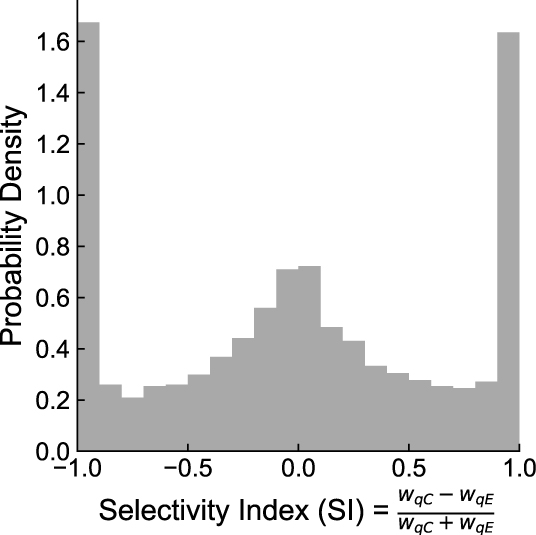

Supplement: Fig S9 [file NIHMS2167012-supplement-Fig_S9.jpg]

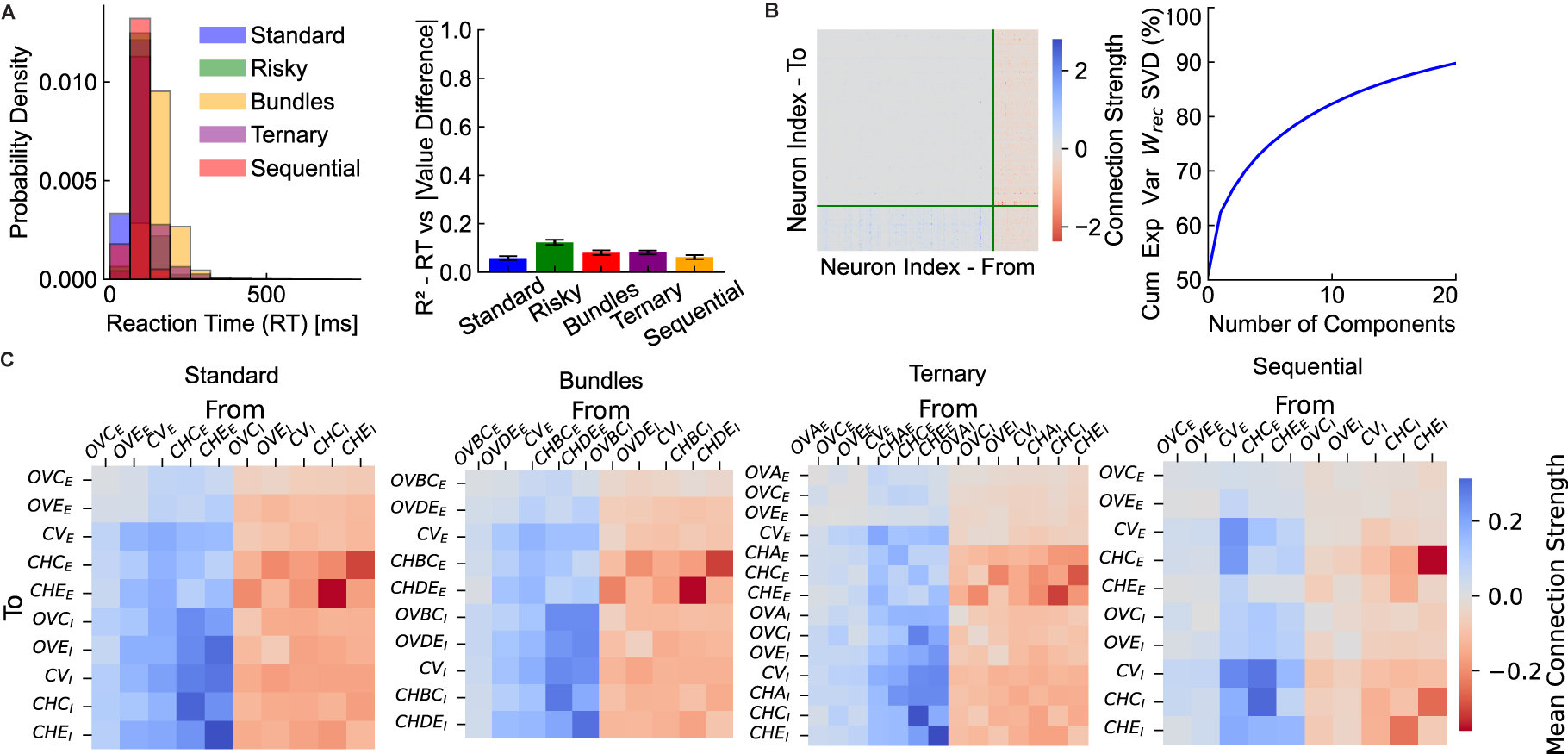

Supplement: Fig S10 [file NIHMS2167012-supplement-Fig_S10.jpg]

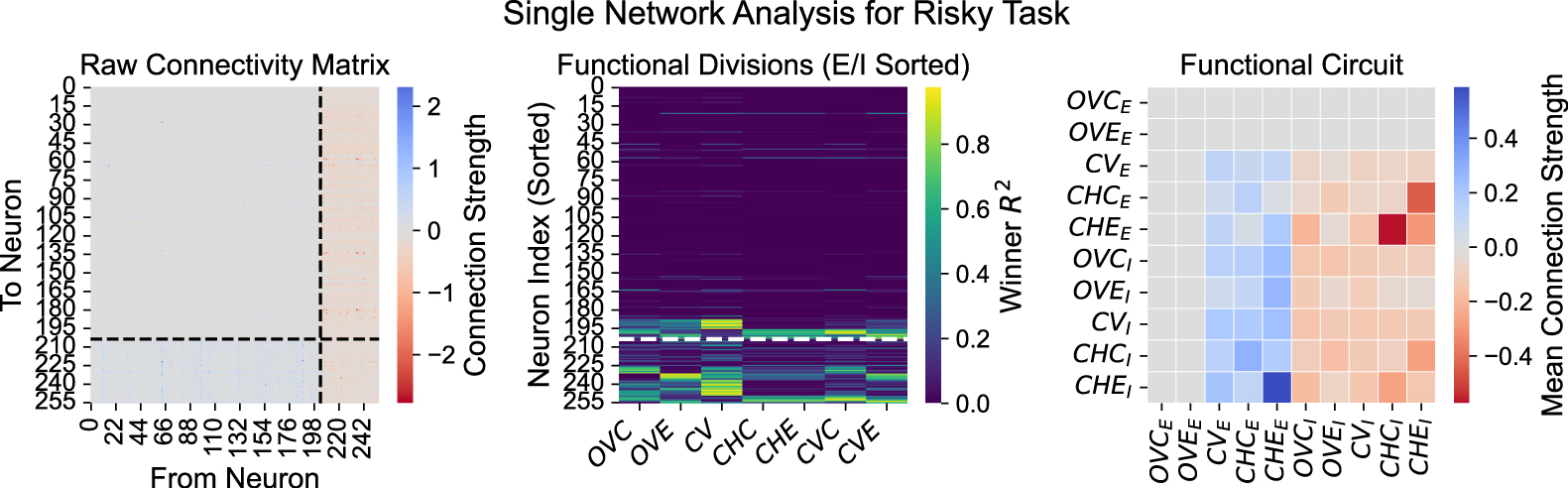

Supplement: Fig S11 [file NIHMS2167012-supplement-Fig_S11.jpg]

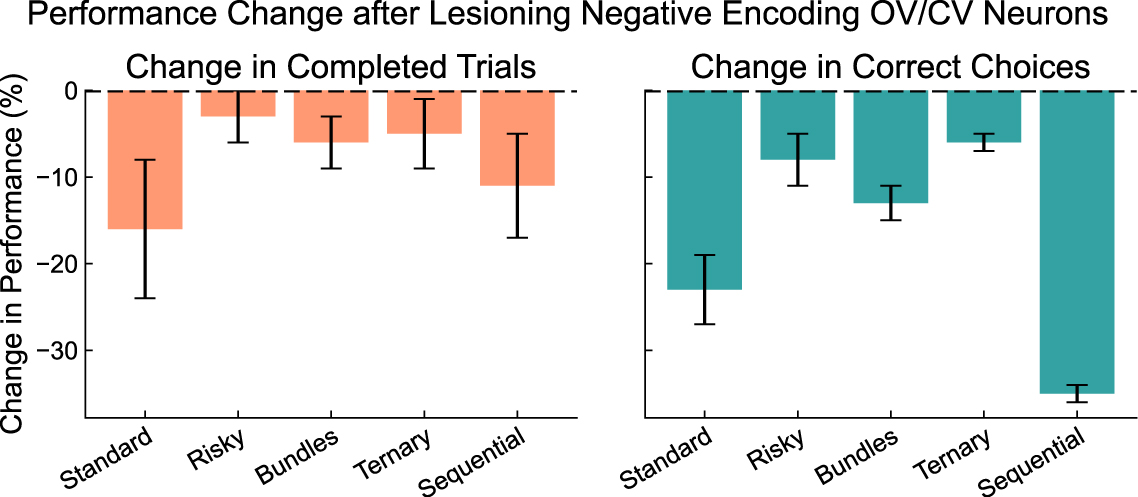

Supplement: Fig S12 [file NIHMS2167012-supplement-Fig_S12.jpg]

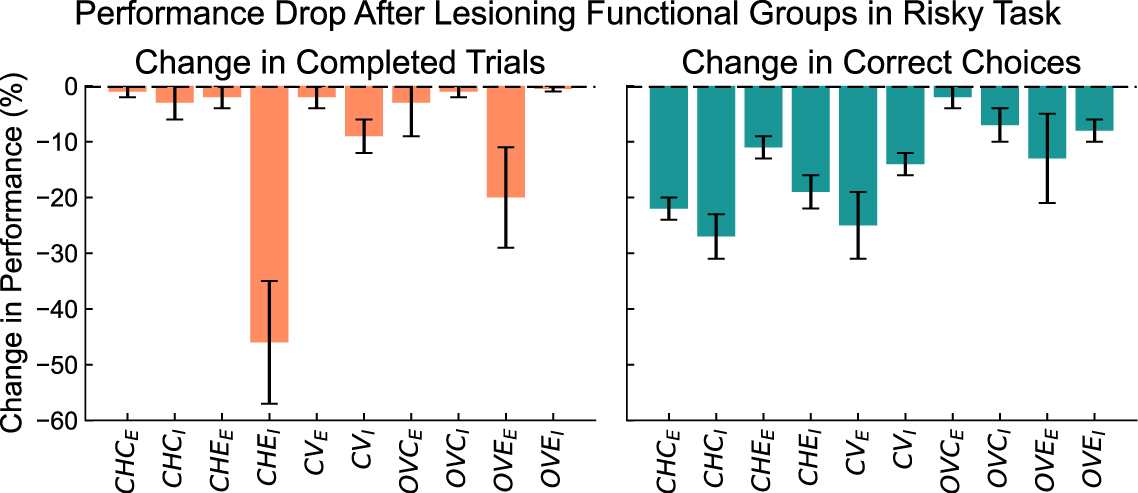

Supplement: Fig S13 [file NIHMS2167012-supplement-Fig_S13.jpg]

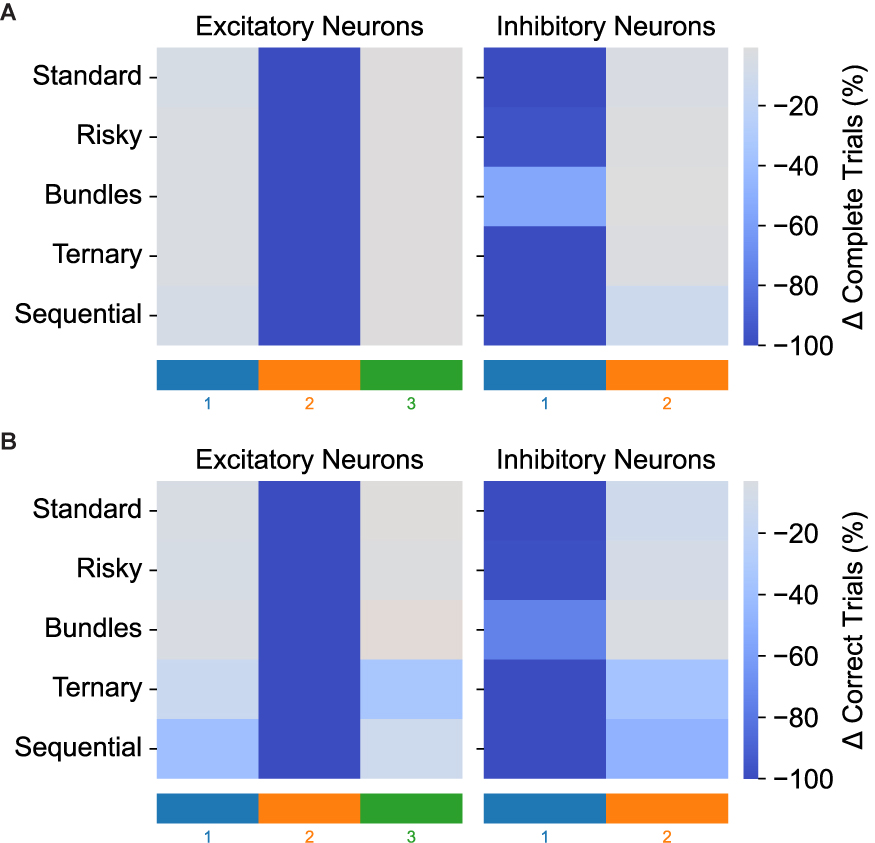

Supplement: Fig S14 [file NIHMS2167012-supplement-Fig_S14.jpg]

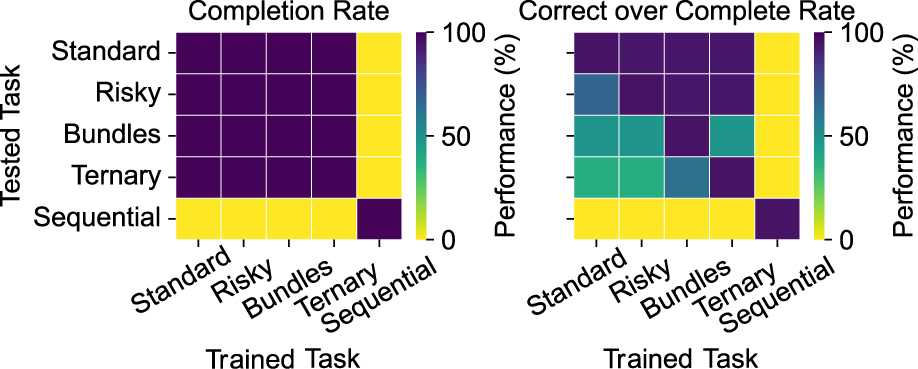

Supplement: Fig S15 [file NIHMS2167012-supplement-Fig_S15.jpg]

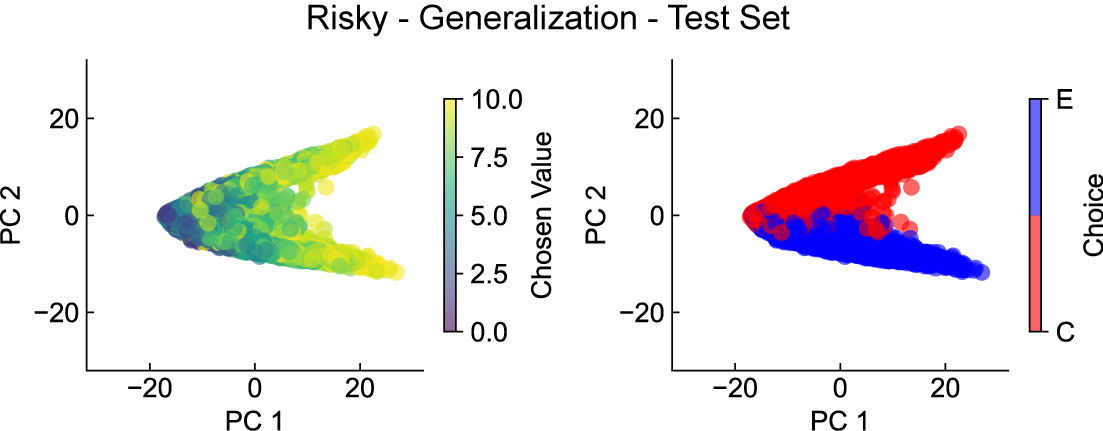

Supplement: Fig S16 [file NIHMS2167012-supplement-Fig_S16.jpg]
